# Perceived Risk, Expected Benefits and Pig Farmers’ Behaviors of Veterinary Drug Usage

**DOI:** 10.3390/ijerph15081716

**Published:** 2018-08-10

**Authors:** Jianhua Wang, Yuanyuan Deng, Hanyu Diao

**Affiliations:** 1 School of Business, Jiangnan University, Wuxi 214122, China; 1080116222@vip.jiangnan.edu.cn; 2 Food Safety Research Base of Jiangsu Province, Jiangnan University, Wuxi 214122, China; 3 College of Economics and Management, China Agricultural University, Beijing 100083, China; yuanyuan.Deng@cau.edu.cn

**Keywords:** perceived risk, expected benefits, pig farmers’ behaviors of veterinary drug usage, self-efficacy

## Abstract

To guarantee the pork quality and safety and the steady development of the pig-breeding industry in China, it is important to control veterinary drugs usage in the pig farming sector. In order to develop an effective intervention that control veterinary drug usage, it is important to perform an in-depth analysis of those factors that can affect the standardized use of veterinary drugs in the pig-breeding process. In this paper, hierarchical regression analysis is used to examine how perceived risk, expected benefits, and self-efficacy influence on the standardized use of veterinary drugs. Data were collected using a multi-stage sampling method from four provinces in China. The results show that expected benefit and self-efficacy have positive impacts on the standardized use of veterinary drugs. Self-efficacy significantly moderated the positive relationships between expected benefits and the negative relationships between perceived risk and standardized use of veterinary drugs.

## 1. Introduction

The pig-breeding industry plays a significant role in China’s animal husbandry industry. In 2015, China’s animal husbandry achieved a total output value of 2.99 trillion yuan, and the pig-breeding industry had an output value of 1.2 trillion yuan, 40% of the total output value [1]. Thus, the pig husbandry is the cornerstone of animal husbandry industry in China. Meanwhile, pork is the primary meat product for the urban and rural residents in China. In 2016, their pork consumption accounted for 70.34% and 82.38% of all meat products respectively [2]. Therefore, the steady development of pig husbandry has much to do with China’s economic growth and social stability. Yet, there are many risks and uncertainties in the pig-breeding process. Now diseases have become a huge threat to the pig husbandry. Diseases harm the pigs’ health and reduce their productivity. Some serious diseases can even kill them and result in direct economic losses. As more production input factors are involved in the pig-breeding process, pig farming has become increasingly a scale-dependent operation, and the pig-breeding density increases. In this context, pig diseases are more and more complicated, hidden, and non-typical, and pose a growing risk, so that the prevention and control of diseases-related risks have become increasingly critical [3]. 

Veterinary drugs play a crucial part in preventing and controlling the diseases-related risks, and they even make contributions to higher agricultural productivity and a steady pork supply. Unfortunately, agricultural producers’ non-compliance with regulations on veterinary drugs in the pig-breeding process generate excessive residues of veterinary drugs and make the pork quality vulnerable to harsh challenges, causing consumers’ physical health to be directly threatened, and also seriously restricting the international competitiveness of China’s pig industry [4]. Thus, to guarantee the pork quality and safety, and the steady development of pig husbandry in China, it is important to control veterinary drugs usage in the pig farming sector.

A number of studies have investigated the determinants of veterinary drug use behavior from different perspectives. Some research suggest that external farm factors are related to antibiotic usage, including labor input [5], housing condition of animals [6]. Other scholars argue that personal variables are the main factors responsible for veterinary drugs usage behavior, for example, gender and age [7,8]. Other factors such as farmers’ risk perception of antibiotic resistance also influence farmers’ antibiotic usage [9]. In order to develop an effective intervention that controls veterinary drugs usage, it is important to perform an in-depth analysis of those factors that can affect the standardized use of veterinary drugs in the pig-breeding process from the perspective of agricultural producers.

There are two problems with existing studies. First, they focus on the influencing factors of antibiotic usage quantity and safe veterinary drugs use behavior, but they rarely probe into the farmers’ choice of standardized use of veterinary drugs. Second, they focused on the examination of individual characteristics, but seldom turn attention to the farmers’ motivation mechanism for their use of veterinary drugs, and fail to dig into the farmers’ key motivations for the use of veterinary drugs. As a matter of fact, the standardized use of veterinary drugs can enhance the pigs’ quality and safety level, but it also generates some costs. Therefore, for those farmers, their standardized use of veterinary drugs may be deemed as their use of new production technology as part of investment decisions. According to Schulz’s rational theory of small farmers, the farmers usually strive for maximum production returns in their allocation of resources and investment in production factors, and their behaviors are the rational choices made after costs, benefits, and risks are weighed. To the best of the authors' knowledge, no study has investigated the effects of expected benefits, perceived risks, and self-efficacy upon the decisions for the use of veterinary drugs in pig farming. Thus, the objective of the paper is to explore the underlying internal characteristics, i.e., expected benefits and perceived risks, on farmers’ behavior when using veterinary drugs and whether these factors are moderated by self-efficacy.

The remainder of this study is organized as follows. Section 2 explains the associated hypotheses and the theoretical framework. Section 3 describes the survey design and measurement of variables. The methodology and results are presented in Section 4. Finally, policy implications are given in Section 5. 

## 2. Hypotheses

### 2.1. Perceived Risks and Standardized Use of Veterinary Drugs

Perceived risk is the possibility of negative results perceived by the farmers in the decision-making process. Some studies reveal that perceived risk is a critical player in agricultural production-related decisions. Trujillo-Barrera (2016) pointed out in the study that perceived risk is a barrier to the adopting sustainable practices, and risk tolerance contributed to a stronger effect of the expected economic benefits on the sustainable productions [10]. Chen, R.J. et al (2009) found in their investigation that some farmers tend to choose a sub-optimal strategy such as self-retained seeds to evade market risks when the seed market is less regulated, and this has a negative effect on the choice of the optimal seeds [11]. Sun, R.Y. and Zhou, J. (2015) mentioned in the study that China’s pig market has not established sound quality safety separation mechanisms, and that the excessive use of veterinary drugs will not have any negative influence on the pigs’ selling price, but will instead drive up the selling price. Thus, one of the reasons for which some agricultural producers tend to make an excessive use of veterinary drugs may be out of the intention to evade the risks arising from the fluctuation of income [12]. Wang and Gu (2013) discovered that some vegetable growers have a trust bias toward whether pesticides, if used according to the pesticide manual, can achieve the desired results. For vegetable growers, this trust bias means that the stipulated use of pesticides may lead to the risks of less than expected effects from their used pesticides, and subsequently to their excessive use of pesticides [13]. In our study, the decisions on the standardized use of veterinary drugs may be faced by the following risks: the return risk—the standardized decisions on the use of veterinary drugs increase the production cost, but fail to gain a high market price; the functional risk—the standardized decisions on the use of veterinary drugs prevent the veterinary drugs from achieving their ideal results; the operational risk—the limited knowledge about the standardized methods or the difficulties involved in the use of these methods may lead to improper operation and even the loss of production; the social risk—standardized use of veterinary drugs decisions is not approved by others. The risk-averse theory that introduces these risk factors to the analysis of the farmers’ economic behaviors assumes that famers are averse to risks, and points out that the farmers’ behavioral logic are not to take risks for higher returns, but to evade risks for guaranteed safety. Therefore, it is hypothesized that:

**Hypotheses 1** **(H1):** *The perceived risk has a negative effect on the agricultural producers’ standardized use of veterinary drugs*.

### 2.2. Expected Benefits and Standardized Use Of Veterinary Drugs

Expected benefits refer to all of the benefits that are expected to be obtained by agricultural producers in the decision-making process, including internal expected benefits and external expected benefits [14]. Zhou (2006) concluded that the vegetable growers can be more active to control the quality safety of vegetables if they are convinced of the income increase, or to gain the psychological satisfaction due to their efforts to control the quality safety of vegetables [15]. In the study of Fraser et al. (2010), the perceived effectiveness of biosecurity measures was related to a farmer’s compliance with those measures [16]. Valeeva et al. (2001) used the Health Belief Model (HBM) to investigate Dutch pig farmers’ perceptions on the risk from pig diseases and the disease risk management strategies, and the research findings showed that the perceived benefits, in term of strategy efficacy, are the most powerful forecast factors in the risk management strategy [17]. The standardized agricultural production can satisfy social expectation and avoid more rigorous regulation. With regard to the standardized use of veterinary drugs, the expected benefits include the premium price of agricultural products, the avoidance of more rigorous regulation, and a better reputation. Those expected benefits will incentivize the agricultural producers to comply with the veterinary drugs usage standard. Therefore, it is hypothesized that:

**Hypotheses 2** **(H2):** *The expected benefits have a positive effect on the agricultural producers’ standardized use of veterinary drugs.*

### 2.3. Self-Efficacy and Standardized Use Of Veterinary Drugs

Self-efficacy means that an individual makes an independent judgment about his or her own ability to achieve the expected result [18], and this judgment then affects his behavioral choices and the efforts made by him [19]. For agricultural producers, self-efficacy refers to judgment of their capabilities to organize and execute courses of action that are required to attain the designated types of agricultural performance, and this judgment can play an important role in their behavioral choice [20]. Therefore, self-efficacy is not a person’s real ability, but his or her evaluation of and confidence in their behavioral ability. Farmers with high self-efficacy believe that they are more competitive, more challenging, more likely to get recognized, more eager to understand agricultural practices, more capable of perceiving the environmental uncertainty, and are more active to grasp new skills. In contrast. Farmers with low self-efficacy may give up their agricultural production in the long run [20]. Wu and Mweemba (2010) found that farmers’ environmental self-efficacy plays a key part in their decision to change their behavior. Specifically, greater perception of one’s capability to improve the environment is significantly associated with a more positive environmental behavior [21]. Ellis-Iversen et al. (2010) discovered that the absence of supportive social norms and of self-efficacy prevent cattle farmers from their intention to control some foodborne diseases [22]. Agricultural producers with low self-efficacy lack confidence in their ability to use veterinary drugs properly, but they also turn more attention to the barriers and risks against the standardized use of veterinary drugs. As a result, they may abandon or postpone the standardized use of veterinary drugs. Agricultural producers with high self-efficacy tend to have stronger self-confidence in their ability to use veterinary drugs properly, and they believe that they can overcome these barriers, and tend to incorporate the standardized use of veterinary drugs. Therefore, it is hypothesized that:

**Hypotheses 3** **(H3):** *Self-efficacy has a positive effect on agricultural producers’ standardized use of veterinary drugs*.

### 2.4. The Moderative Role Of Self-Efficacy

In all the existing studies on the relation between the perceived risk, expected benefits and the behavioral decision-making, most scholars have paid little attention to the fact that the perceived risk and expected benefits are an individual’s subjective expectations, and that their effects are bound to be moderated by individual differences, such as self-efficacy [23]. Stumpf et al. (1987) found that those individuals with high self-efficacy tend to adopt an active response strategy orientated by problems, but those individuals with low self-efficacy tend to adopt a passive response strategy oriented by sentiment [24]. The problem-oriented response strategy can help individuals to relieve pressures noticeably. These existing studies have revealed that self-efficacy could effectively relieve the negative effects of pressures over individuals. For agricultural producers with high self-efficacy, the perceived risk may impose a limited effect on the standardized use of veterinary drugs. These individuals have sufficient knowledge and skills concerning the standardized use of veterinary drugs. When confronted by the high perceived risk, they will more actively respond to uncertain results and risks. However, for agricultural producers with low self-efficacy, the perceived risk may impose a strong effect on the standardized use of veterinary drugs. Because of their insufficient knowledge and skills, they will lack confidence in behavioral decisions and will tend to give up halfway instead of seeking solutions when negative factors happen. For agricultural producers with high self-efficacy, the expected benefits may impose a strong effect on the standardized use of veterinary drugs. When confronted by highly expected benefits, they will further strengthen their expectations about success. However, for agricultural producers with low self-efficacy, the expected benefits may impose a small effect on the standardized use of veterinary drugs. These individuals tend to reduce their expectations about success. Therefore, it is hypothesized that.

**Hypotheses 4** **(H4):** *Self-efficacy plays a negative moderation role when the perceived risk affects the agricultural producers’ standardized use of veterinary drugs*.

**Hypotheses 5** **(H5):** *Self-efficacy plays a positive moderation role when the expected benefits affect the agricultural producers’ standardized use of veterinary drugs*.

**Figure 1:**
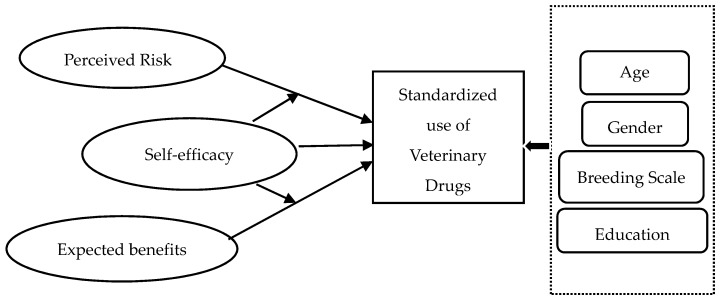
Conceptual framework.

To conclude, the framework of theoretical analysis is constructed, as indicated in Figure 1.

## 3. Design of Study

### 3.1. Sources of Data

The data used in this study mainly came from the survey organized in January and February 2017. The survey covered related information in 2016. Top attention was paid to those provinces with high pig-breeding density, such as Henan and Shandong. To gain a general picture of pig farmers’ decisions on veterinary drugs across major pig-breeding regions of China, the related regions in Jiangxi and Guizhou were sampled so that the collected data here could be more representative. The survey data used here were collected through stratified random sampling. First, the sample size for the Phase 1 sample unit was determined. Based on the study conducted by Hou and Huo (2016) [25], the specific sample size was calculated as follows:

(1) $$\;n_{1}=\left(\frac{u_{\alpha }v}{1-p_{c}}\right)\;$$

**Figure 2:**
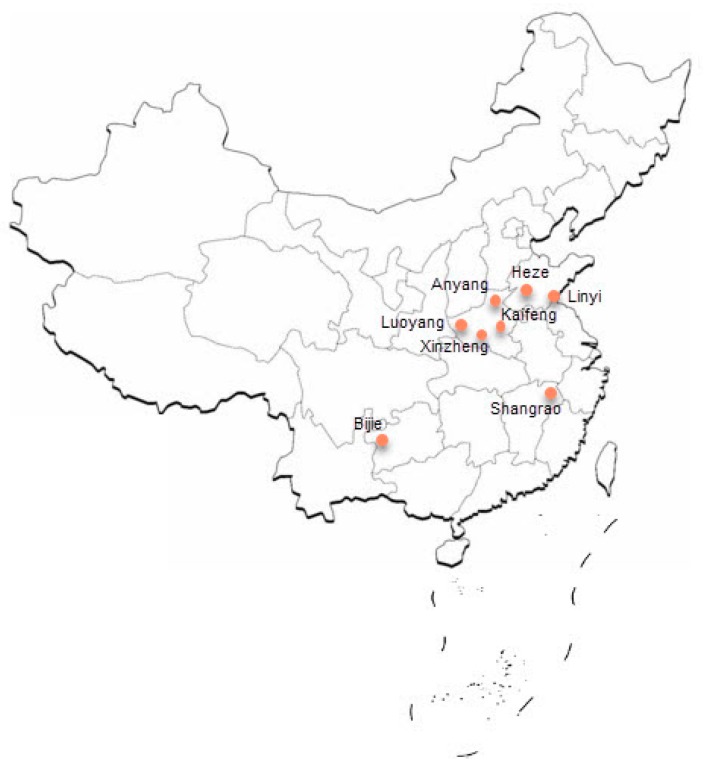
Location of the study area.

In Equation (1), α=0.01 is the value of $u_{\alpha }$ when the estimated reliability is described as α=0.05; v is the coefficient of variation, usually about 0.3, but here 0.25, $p_{c}$ is the estimated sampling precision, and here is 0.8. It can thus be concluded that $n_{1\;}=6$. To guarantee the sampling precision, the Phase 1 sample size should be greater than 6. The probability proportionate to size (PPS) sampling method was used. The sample cities thus determined included Luoyang, Kaifeng, Xinzheng, Anyang County, Heze, Linyi, Bijie, and Shangrao (see Figure 2). Then, the Phase II sample size (number of pig farmers) was calculated as follows:

(2) $$\;n_{2}=\frac{z_{\alpha }p\left(1-p\right)}{\Delta^{2}}\;$$

In Equation (2), if α=0.01,${\;Z}_{a}=2.576$. The percentage variance p(1−p) was replaced by the maximum value 0.25, and the sampling error was kept within 5%. As a result, the sample size of agricultural producers was at least 275. The sampling method of the pig farmers is specified as follows. Several villages (towns) were sampled for each sample, and then a random selection of pig farmers was conducted for each sampled village (town). 

To guarantee the validity of this questionnaire, a small-scale pilot survey was conducted in Xinzheng city, Henan Province before the formal survey was initiated. Some implicit, ambiguous or respondent-averse questions were then modified. To ensure the authenticity and reliability of the survey results, training was organized for the investigators before the formal survey was started. The field survey took the form of an interview and a questionnaire, and the respondents were the family members that directly affected the pig-breeding decisions, to guarantee the accuracy and the response rate of the questionnaires. This survey collected 480 questionnaires, including 397 valid questionnaires if those questionnaires with absence of key data and untrue answers were dismissed. Regionally speaking, Henan Province’s sample size was 194, 49% of total samples; Shandong Province’s sample size was 128, 32% of total samples; Jiangxi Province’s sample size was 41, 10% of total samples; Guizhou Province’s sample size was 34, 9% of total samples.

### 3.2. Descriptive Statistics

Table 1 mainly describes the basic statistics of samples. Among the 397 farmers, 59.2% were male and 40.8% were female. If sampling was sufficiently random, this figure would indicate that the agricultural producers engaged in pig breeding were mainly male. When it came to age structure, the pig farmers were mainly aged from 41 to 60. Specifically, the respondents aged 41–50 constitute as much as 44.8% of the total sample, and the respondents aged 51–60 accounted for 27.7%. This means that the agricultural producers engaged in pig breeding were mainly middle-aged and old people. The farmers’ education was examined, and those with a junior high school education and below made up 81.4%. Of the survey samples, the small-scale pig-breeding households constituted 27.7%, and the professional pig-breeding households 7.8%. The pig-breeding households differed in their scales and the samples were rationally distributed. Additionally, the data about the large-scale pig-breeding households were not easily obtained in the survey, so this type of pig-breeding households accounted for a small ratio of the sample. It is necessary to point out that the pig-breeding incomes only made up a small share of the total incomes in the pig-breeding households. Specifically, the pig-breeding households whose pig-breeding incomes were less than 50% of their total incomes constituted 43.8% of all the pig-breeding households. A total of 68.8% pig-breeding households had other incomes sources than pig breeding.

### 3.3. Description about Variables

This study involves four key variables, including perceived risk, expected benefits, self-efficacy, and the standardized use of veterinary drugs. Each dimension has three to six options, and involves 20 questions. The scoring scale is a Likert 5-scale, which ranges from 1 point (strongly disagree/strongly ignorant) to 5 points (strongly agree/strongly knowledgeable). The questionnaires were designed after the research findings of domestic and foreign scholars were analyzed. Some small interviews were then organized to make modifications to less perfect and explicit questions and options. Some details were provided about the measurement of all the variables. The perceived risk mainly involved four dimensions, such as the return-risk, the functional risk, the operational risk, and the social risk. The measurement was mainly based on the research ideas of Ashari et al. (2016) [26], and Jacoby and Kaplan (1972) [27], and modified the specific questions. The expected benefits included economic benefits, psychological benefits, and policy benefits. The design of the scoring scale was based on the studies by Ashari et al. (2016) [26] and Visschers et al. (2014) [28]. The measurement was mainly based on Roy’s study (2009) [20], and modified the specific questions. The standardized use of veterinary drugs involved three dimensions, such as the use of veterinary drugs in strict accordance with the specification of veterinary drugs, and with the regulations on the withdrawal period, and with the intention to conduct the residue detection of veterinary drugs or the entrusted detection. The measurement was based on Sun’s selection of variables (2014) [29]. Refer to Table 2 for the description about the measurement of variables.

Based on the related literature, this study uses the individual characteristics and production and operation characteristics that affect the use of veterinary drugs as the model’s control variables, including gender [7], age [8], education [4], and breeding scale [30]. Refer to Table 3 for the details about variable assignments and descriptive statistics.

### 3.4. Reliability and Validity Testing

Reliability is mainly used to measure the reliability, consistency, and stability of a questionnaire or scale. The reliability of scale in this study is tested through Cronbach’s alpha (α) coefficient. In DeVellis’s study (1991) [31], this coefficient should be no less than 0.65, and should be preferably more than 0.7. In Table 1, the coefficients for all the variables are over 0.65. This implies that the scale’s reliability was good. Validity is mainly used to determine whether the measurement results of the designed scale can give relevant insights into the targeted questions. Validity is usually divided into convergent validity and discriminant validity. In Table 2, the factor loadings for all of the measured questions are over 0.6. This means that the study variables in this study had a satisfactory convergent validity. In Table 4, the correlation coefficient between two variables is less than 0.85. This shows that the designed scale in this study had discriminant validity to some degree [32]. 

## 4. Empirical Analysis

### 4.1. Correlation Analysis

Refer to Table 4 for the correlation coefficient between every two main variables. According to this table, the self-efficacy and expected benefits were positively correlated to the standardized use of veterinary drugs. This offered preliminary support for the related hypothesis in this study. The hypothesis of the study was subject to the detailed test as below.

### 4.2. Hypotheses Testing

The analysis method in this study was the hierarchical regression analysis. When the hierarchical regression analysis is made, the regression model is created by taking the following steps: only control variables are included in the Model 1, then the main variables and the moderator variables are included in turn, and finally, the interactive items of the main variables and moderator variables are included. As the interactive items are introduced, the related variables are subject to the Zero-centered to avoid the multi-colinearity possibly found in the regression model [33]. In this way, the result of the hierarchical regression analysis as indicated in Table 5 can be obtained.

As indicated in Model 1, the agricultural producer’s breeding scale and age exerted a prominent effect on the standardized use of veterinary drugs. Specifically, the larger breeding scale, the stronger the intention to make standardized use of veterinary drugs (β = 0.076, *p* < 0.05). This conclusion was similar to the research findings by Sun and Zhou (2015) [12]. According to their research findings, the pig-breeding scale had a considerably negative effect on the farmers’ excessive use of veterinary drugs. These two scholars further explained that a smaller pig-breeding scale means a more arbitrary attitude toward the control of diseases. Moreover, there is another possible explanation that a larger pig-breeding scale means a stronger dependence on pig breeding and a larger possibility of using new breeds and management technologies. This will contribute to the successful implementation of the standardized use of veterinary drugs. In contrast, age exerted a negative effect on the standardized use of veterinary drug (β = −0.094, *p* < 0.05). This may be attributed to the fact that the pig-breeding experience becomes more extensive along with age. The decisions on the use of veterinary drugs are highly experience-dependent, so not enough attention is paid to the series of requirements for the standardized use of veterinary drugs. 

According to Model 2, the expected benefits exerted a significant impact upon the use of veterinary drugs. Therefore, H2 was supported. This means that their standardized use of veterinary drugs can be encouraged by increasing the expected benefits. In contrast, the perceived risk produced a limited effect on the standardized use of veterinary drugs. The results did not support previous findings that perceived risk is an important factor regarding antibiotic usage [28]. According to this study, it is because agricultural producers consider the standardized use of veterinary drugs not as something risky, but as something that meets long-term interests. Now many agricultural producers arbitrarily use veterinary drugs. This may be related to their strong path-dependence in the use of veterinary drugs. A larger production cost is necessary before this drug-using habit is changed. In fact, the habit usually remains intact for the sake of benefits. According to Model 3, self-efficacy exerted a positive impact upon the standardized use of veterinary drugs (β = 0.250, *p* < 0.001). This means that the agricultural producers’ standardized use of veterinary drugs was restricted by low self-efficacy.

According to Model 4, self-efficacy positively moderated the effect of the expected benefits on the standardized use of veterinary drugs. This implies that the higher self-efficacy, the stronger effect of the expected benefits on the standardized use of veterinary drugs. Besides, self-efficacy can negatively regulate the effect of the perceived risk on the standardized use of veterinary drugs. This means that the stronger the self-efficacy, the weaker an effect by the perceived risk on the standardized use of veterinary drugs. Agricultural producers with high self-efficacy have a stronger ability to deal with pig immunization, and disease diagnosis and treatment, and have a more standardized use of veterinary drugs in the light of the type, quantity, and form of use, to achieve better results for pig immunization, and disease diagnosis and treatment. In other words, the strong ability to control in the use of veterinary drugs increased the positive incentive effect of the expected benefits to some degree, and reduced the negative inhibitive effect of the perceived risk. For the agricultural producers with low self-efficacy, the positive incentive effect of the expected return was not noticeable, but the negative inhibitive effect of perceived risk was distinct. This was similar to the research findings by Wang and Liu (1996) [34]. They discovered that ignorance about technical contents and effects caused farmers in many poverty-stricken areas to face huge subjective risks in their technical decisions. As a result, they may abandon or postpone the use of new technologies. Therefore, it is suggested that, in addition to the applicable technologies, the poorer farmers should grasp as much technological information as possible, the new technologies should be made less uncertain for farmers, and farmers’ subjective risks of self-assessment should be mitigated to ensure the successful communication of new technologies in the poverty-stricken areas.

## 5. Conclusions

On one hand, the results show that expected benefits have a positive influence on agricultural producers’ standardized use of veterinary drugs. It is advisable to increase the expected benefits for agricultural producers’ standardized use of veterinary drugs, e.g. to introduce and improve the food quality signal identification system, and differentiate the pork quality indicators through price differences, so as to increase the economic benefits of the standardized use of veterinary drugs, to organize the compulsory testing of pork products sold in the market, to increase the market access threshold, to tighten the linkage between monitoring and law enforcement, and to investigate the agricultural products with quality safety threats and punish the related production operators according to the law, in order to raise the policy benefits of the standardized use of the veterinary drug. On the other hand, agricultural producers’ self-efficacy plays a significant role in standardized use of veterinary drugs. This result suggests that the knowledge and technological training for agricultural producers should be organized at different levels, through different channels, and in different forms, to constantly enhance self-efficacy in the standardized use of veterinary drugs.

## Figures and Tables

**Table 1:** Basic statistics of samples.

Statistical Characteristic	Classification Indicator	Sample Size	Percentage (%)
Gender	Male	235	59.2
	Female	162	40.8
Age	20–30	15	3.8
	31–40	77	19.4
	41–50	178	44.8
	51–60	110	27.7
	≥61	17	4.3
Educational level	Primary School or below	106	26.7
	junior middle school	217	54.7
	High school and secondary school	68	17.1
	junior college and above	6	1.5
Non-Farming Income	Yes	273	68.8
	No	124	31.2
Percentage of Pig-Breeding Incomes in Family Total Income	≤30%	131	33
	31–50%	43	10.8
	51–80%	98	24.7
	≥81%	125	31.5
Breeding Scale	<50 hogs	110	27.7
	50–500 hogs	256	64.5
	>500 hogs	31	7.8

**Table 2:** Core variable description and measurement.

Variable	Item	Factor Loading	Cronbach’ α
Independent Variable			
Expected Benefits	Standardizing the use of veterinary drugs can meet the requirements of pig buyers	0.802	0.779
	Standardizing the use of veterinary drugs is safe and risk-free	0.772	
	Standardizing the use of veterinary drugs is a necessary production process for the production of high-quality pork	0.749	
	Standardizing the use of veterinary drugs is beneficial to consumers' health and avoids product safety risks	0.708	
	Standardizing the use of veterinary drugs, the market price of live pigs is high	0.523	
	Standardizing the use of veterinary drugs can avoid more stringent controls	0.680	
Perceived Risk	You are concerned that regulating the use of veterinary drugs is not acceptable to most farmers	0.780	0.717
	You are concerned that the regulatory use of veterinary drugs is not effective in preventing the risk of pig disease	0.714	
	You are worried that regulating the use of veterinary drugs will put your benefits at risk	0.666	
	You are concerned about the difficulty of regulating the use of veterinary drugs	0.657	
Moderator Variable			
Self-efficacy	Your knowledge of the effects of veterinary drugs	0.823	0.800
	Your knowledge of pig disease	0.780	
	According to your ability, it is unlikely that there will be any disease in the process of raising pigs	0.686	
	You can easily obtain the operation standard information for pig breeding	0.664	
	You are proficient in the use of veterinary medicine technology and knowledge	0.611	
Dependent Variable			
Behavioral Selection	You comply with the regulations on the withdrawal period of veterinary drugs	0.743	0.670
	You strictly follow the regulation of a rest period in the process of breeding	0.730	
	You will conduct regular veterinary drug residue testing or commission monitoring	0.669	

**Table 3:** Variable assignments and descriptive statistics.

Variable	Assignment Instructions	Mean Value	SD	Minimum Value	Maximum Value
Gender	Male = 1; Female = 0	0.408	0.492	0	1
Age	20–30 = 1; 31–40 = 2; 41–50 = 3; 51–60 = 4; ≥60 = 5	3.093	0.887	1	5
Educational Level	Primary School or Below = 1; Junior high school = 2; High school and secondary school = 3; Junior college = 4; Graduate and above = 5	1.945	0.747	1	5
Breeding Scale	Size class (<50 = 1; 50–100 = 2; 100–200 = 3; 200–500 = 4, >500 = 5)	1.980	1.068	1	5
Perceived Risk	The arithmetical average of earnings risk, functional risk, operational risk, and social risk	1.720	0.556	1.250	5.000
Expected Benefits	The arithmetic mean values of economic, psychological, and policy gains	3.400	0.686	1.667	5.000
Self-efficacy	The arithmetic mean of each item	2.845	0.466	1.800	5.000
Behavioral Selection	Standardized the use of veterinary drugs three dimensional arithmetic mean values	2.147	0.699	1.000	5.000

**Table 4:** Descriptive statistics.

Variable	Perceived Risk	Self-Efficacy	Expected Benefits	Drug Usage	Gender	Age	Educational Level
Perceived risk	1						
Self-efficacy	−0.101						
Expected Benefits	−0.122 *	0.174 **					
Drug Usage	−0.064	0.335 **	0.258 **				
Gender	−0.020	−0.033	−0.021	0.060			
Age	0.066	−0.091	−0.184 **	−0.127 *	0.036		
Educational Level	−0.077	−0.044	0.081	0.089	−0.033	−0.207 **	
Breeding scale	0.015	−0.008	0.061	0.115^*^	0.020	0.000	−0.038

** and * respectively represent significance at 5% and 10% statistical levels.

**Table 5:** Result of hierarchical regression analysis.

Variables	Model 1	Model 2	Model 3	Model 4
Gender	0.092	0.096	0.109	0.101
Age	−0.094 *	−0.060	−0.042	−0.050
Education Level	0.108	0.089	0.126	0.091
Breeding Scale	0.076 *	0.066 *	0.070 *	0.070 *
Perceived Risk	−	−0.101	−0.097	−0.088
Expected Benefits	−	0.236 ***	0.187 ***	0.177 ***
Self-efficacy	−	−	0.267 ***	0.250 ***
Perceived risk × Self-efficacy	−	−	−	0.073 **
Expected Benefits × Self-efficacy	−	−	−	0.044 *
*R* ^2^	0.038	0.092	0.181	0.378
Adjusted *R*^2^	0.027	0.076	0.164	0.354
*F* value	3.491 **	5.871 ***	10.957 ***	20.499 ***

***^,^ ** and * respectively represent significant statistical levels of 1%, 5% and 10%.
